# Inhibitory Effects of Momordicine I on High-Glucose-Induced Cell Proliferation and Collagen Synthesis in Rat Cardiac Fibroblasts

**DOI:** 10.1155/2018/3939714

**Published:** 2018-10-08

**Authors:** Po-Yuan Chen, Neng-Lang Shih, Wen-Rui Hao, Chun-Chao Chen, Ju-Chi Liu, Li-Chin Sung

**Affiliations:** ^1^Department of Biological Science and Technology, College of Biopharmaceutical and Food Sciences, China Medical University, Taichung 40402, Taiwan; ^2^Department of Life Sciences, College of Science, National University of Kaohsiung, Kaohsiung 811, Taiwan; ^3^Division of Cardiology, Department of Internal Medicine, Shuang Ho Hospital, Taipei Medical University, New Taipei City 23561, Taiwan; ^4^Department of Internal Medicine, School of Medicine, College of Medicine, Taipei Medical University, Taipei 11031, Taiwan

## Abstract

Diabetes-associated cardiac fibrosis is a severe cardiovascular complication. Momordicine I, a bioactive triterpenoid isolated from bitter melon, has been demonstrated to have antidiabetic properties. This study investigated the effects of momordicine I on high-glucose-induced cardiac fibroblast activation. Rat cardiac fibroblasts were cultured in a high-glucose (25 mM) medium in the absence or presence of momordicine I, and the changes in collagen synthesis, transforming growth factor-*β*1 (TGF-*β*1) production, and related signaling molecules were assessed. Increased oxidative stress plays a critical role in the development of high-glucose-induced cardiac fibrosis; we further explored momordicine I's antioxidant activity and its effect on fibroblasts. Our data revealed that a high-glucose condition promoted fibroblast proliferation and collagen synthesis and these effects were abolished by momordicine I (0.3 and 1 *μ*M) pretreatment. Furthermore, the inhibitory effect of momordicine I on high-glucose-induced fibroblast activation may be associated with its activation of nuclear factor erythroid 2-related factor 2 (Nrf2) and the inhibition of reactive oxygen species formation, TGF-*β*1 production, and Smad2/3 phosphorylation. The addition of brusatol (a selective inhibitor of Nrf2) or Nrf2 siRNA significantly abolished the inhibitory effect of momordicine I on fibroblast activation. Our findings revealed that the antifibrotic effect of momordicine I was mediated, at least partially, by the inhibition of the TGF-*β*1/Smad pathway, fibroblast proliferation, and collagen synthesis through Nrf2 activation. Thus, this work provides crucial insights into the molecular pathways for the clinical application of momordicine I for treating diabetes-associated cardiac fibrosis.

## 1. Introduction

The prevalence of diabetes mellitus (DM) is rapidly increasing, and cardiovascular complications are the principal causes of morbidity and mortality among patients with DM. The Framingham study reported a 2.4-fold increase in the incidence of heart failure in diabetic men and a 5-fold increase in diabetic women [1, 2]. Diabetic cardiomyopathy (DCM), which was first introduced in 1972 by Rubler et al., is one of the most severe irreversible complications of DM [3]. DCM is characterized by myocardial fibrosis, left ventricular hypertrophy, and compromised left ventricular systolic and diastolic functions [2, 4, 5]. Left ventricular diastolic dysfunction is a major characteristic of DCM, and recent studies revealed that over 50% of asymptomatic diabetic patients have diastolic dysfunction [1, 6]. Cardiac fibroblasts are enmeshed in the myocardium and play a crucial role in maintaining the integrity and homeostasis of the interstitial matrix in the adult heart [7–9]. In patients with diabetes, profibrotic myofibroblasts are mainly differentiated from cardiac fibroblasts, resulting in inefficiently organized fibrotic matrices, which can lead to myocardial fibrosis, stiffness, and diastolic dysfunction [4]. However, the mechanisms involved in the pathological changes of DCM are not completely understood. Because fibrosis plays a critical role in the pathology of DCM, we focused on the molecular mechanism involved in cardiac fibrosis in DM [5, 10].

Oxidative stress may play a fundamental role in inducing cardiomyopathy in patients with chronic DM [5, 11–14]. Various studies have demonstrated that DM is associated with increased formation of reactive oxygen species (ROS) and a decrease in antioxidant potential [14, 15]. An animal study revealed that ROS could directly induce fibrosis by promoting fibroblast proliferation and collagen synthesis in the setting of DM [15]. The transcription factor nuclear factor erythroid 2-related factor 2 (Nrf2), a basic leucine zipper protein, plays a fundamental role in antioxidant response element- (ARE-) dependent heme oxygenase-1 (HO-1) gene expression. HO-1 is considered a critical endogenous antioxidant and cytoprotective enzyme, which may be upregulated by Nrf2 [16]. The Nrf2/HO-1 signaling pathway has been recognized as the most crucial cellular defense mechanism against oxidative stress [17].

Transforming growth factor-*β*1 (TGF-*β*1) is involved in cardiac fibrosis in DCM [4, 10, 18, 19]. Small mothers against decapentaplegic (Smad) is an intracellular signal transduction protein of the TGF-*β*1 pathway and is involved in these pathological changes [19]. ROS can induce TGF-*β*1 activation, thus resulting in cardiac fibrosis. Euler demonstrated a reduction in TGF-*β*1 levels in patients treated with N-acetylcysteine (NAC) [20].

**Figure 1:**
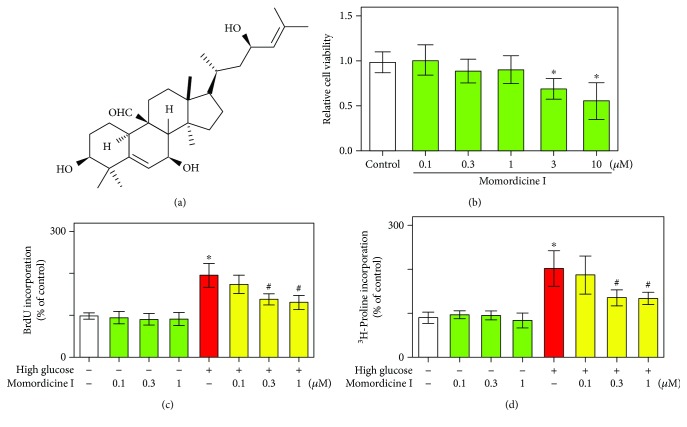
Effects of momordicine I on high-glucose-induced cell proliferation and collagen synthesis in rat cardiac fibroblasts. (a) Chemical structure of momordicine I. (b) Effect of momordicine I (0.1, 0.3, 1, 3, and 10 *μ*M) on cell viability in cardiac fibroblasts. Cell viability was quantified through MTT assay. Effects of momordicine I on high-glucose-stimulated fibroblast proliferation (c) and collagen synthesis (d) were assessed through BrdU and ^3^H-proline incorporation. Rat cardiac fibroblasts were cultured in a serum-free normal-glucose medium (5.6 mM glucose) or high-glucose medium (25 mM glucose) in the absence or presence of momordicine I (0.1, 0.3, and 1 *μ*M) for 24 h. Results were presented as mean ± SEM (*n* = 4). ^∗^*P* < 0.05 versus the control group; ^#^*P* < 0.05 versus the high-glucose group.

Clinical studies have demonstrated that hyperglycemia is a crucial etiologic factor leading to diabetic complications [21]. Diastolic dysfunction is correlated with the degree of hyperglycemia; however, intensive glucose-lowering therapy does not always reduce the risk of DCM [1, 22]. Oxidative stress may be a target for intervention. Other than DM drugs, *Momordica charantia* (bitter gourd or bitter melon), a member of the Cucurbitaceae family, has been used to manage hyperglycemia and the early signs of DM since ancient times in some countries [23–26]. Momordicine I (Figure 1(a)), a bioactive saponin and cucurbitane-type triterpenoid, has been isolated from *Momordica charantia* [27–29]. Momordicine I was reported to be a beneficial natural source of antioxidants [30]. Treatment with momordicine I might have therapeutic potential in improving diabetic myocardial dysfunction. However, little information is available about the effect of momordicine I on the cell proliferation and collagen synthesis of cardiac fibroblasts induced by high glucose.

Thus, we hypothesized that momordicine I is particularly capable of suppressing high-glucose-induced cell proliferation and collagen synthesis in cardiac fibroblasts and therefore decelerates the progression of DCM. The specific aims of this study are to delineate how momordicine I inhibits high-glucose-induced cardiac fibroblast activation through the modulation of ROS, Nrf2/HO-1 pathway, and TGF-*β*1/Smad pathway.

## 2. Material and Methods

### 2.1. Antibodies and Reagents

Dulbecco's modified Eagle's medium (DMEM), fetal calf serum, and tissue culture reagents were obtained from Invitrogen/GIBCO (Grand Island, NY, USA). Momordicine I (>99% purity, kindly provided by Dr. Shi-Yie Cheng, Department of Life Sciences, College of Science, National University of Kaohsiung, Kaohsiung, Taiwan, ROC) was dissolved in dimethyl sulfoxide (DMSO), and the DMSO content in all groups was 0.1%. Anti-p-Smad2/3, anti-Smad2/3, anti-GAPDH, and anti-PARP antibodies were purchased from Cell Signaling Technology (Boston, MA, USA). Anti-HO-1 and anti-Nrf2 antibodies were purchased from Santa Cruz Biotechnology (Santa Cruz, CA, USA). Brusatol, the Nrf2-specific inhibitor, and all other reagent-grade chemicals were acquired from Sigma-Aldrich Chemical Co. (St. Louis, MO, USA).

### 2.2. Culture of Rat Cardiac Fibroblasts

Primary cultures of neonatal rat cardiac fibroblasts were isolated from the hearts of 1-day-old Sprague–Dawley rat pups as previously described [8]. The investigation conformed to the Guide for the Care and Use of Laboratory Animals published by the US National Institutes of Health (NIH publication no. 85–23, revised 1996) and was approved by the Institutional Animal Care and Use Committee of Taipei Medical University. Cardiac fibroblasts grown in culture dishes from the second to fourth passages were used in the experiments and were >99% positive for vimentin antibodies (Sigma-Aldrich). Serum-containing medium from the cultured cells was replaced with serum-free medium, and the cells were subsequently exposed to the treatments as indicated. For fibroblast proliferation assay and TGF-*β*1 secretion assay, following incubation with momordicine I for 12 h, cardiac fibroblasts were then exposed to a serum-free normal-glucose medium (5.6 mM glucose) or high-glucose medium (25 mM glucose) for another 24 h before analyses.

### 2.3. 3-(4,5-Dimethylthiazol-2-yl)-2,5-diphenyltetrazolium Bromide (MTT) Assay

MTT assay was used to measure cell viability. After 24 h incubation with various concentrations of momordicine I as indicated, MTT (50 *μ*L) was added and cells were cultured for additional 4 h. Subsequently, cells were lysed using DMSO and the absorbance was measured at 490 nm by a spectrophotometer.

### 2.4. Proliferation Assay

Proliferation was assessed through quantifying 5-bromo-2′-deoxyuridine (BrdU) incorporation [7]. Cells were removed from the culture dishes by adding trypsin and were subsequently centrifuged. Cell proliferation was assessed through the incorporation of BrdU. BrdU incorporation was determined using a cell proliferation enzyme-linked immunosorbent assay (ELISA) kit (Roche Diagnostics, Mannheim, Germany) in accordance with the manufacturer's instructions.

### 2.5. ^3^H-Proline Incorporation Assay

Cardiac fibroblasts were incubated using ^3^H-proline (1 mCi/well, L-[2,3,4,5-^3^H]-proline; Amersham Biosciences, Little Chalfont, Buckinghamshire, United Kingdom) for 24 h; subsequently exogenous ^3^H-proline incorporation was determined through scintillation counting.

### 2.6. Measurement of TGF-*β*1 Concentrations

TGF-*β*1 levels were measured in a culture medium using a commercial ELISA kit (R&D Systems Inc., Minneapolis, MN, USA) in accordance with the manufacturer's protocols. Results were normalized to cellular protein content in all experiments and expressed as percentages relative to the control group.

### 2.7. Western Blot Analysis

Nuclear proteins were prepared as previously described [31]. Western blot analysis was performed in accordance with a previously described method [8]. Whole-cell extracts were obtained in a radioimmunoprecipitation assay buffer (Roche Diagnostics GmbH, Germany). Extracts or proteins were separated through sodium dodecyl sulfate polyacrylamide gel electrophoresis followed by electrotransfer to polyvinylidene difluoride membranes and probed with antisera before the introduction of horseradish peroxidase-conjugated secondary antibodies. The proteins were visualized through chemiluminescence in accordance with the manufacturer's instructions (Pierce Biotechnology Inc., Rockford, IL, USA). Images were quantified through densitometry. The densitometry for each band of the phosphorylated form of specific protein (e.g., Smad2/3), the total level of specific protein (e.g., Smad2/3), and loading control (e.g., GAPDH) were measured separately. The specific nonphosphorylated total protein (e.g., Smad2/3) was then normalized against the loading control (e.g., GAPDH) and used the normalized total protein for normalization of the specific phosphoprotein (e.g., Smad2/3). Each control group's value was used to normalize the values of the individual groups obtained in the same experiment. Independent values of control data were normalized on the control mean value, with the control mean value equal to 1, to generate control values in the bar graph.

### 2.8. Flow Cytometric Assay of 2′,7′-Dichlorodihydrofluorescein Oxidation

Intracellular ROS production was determined based on the oxidation of 2′,7′-dichlorodihydrofluorescein (DCFH) to a fluorescent 2′,7′-dichlorofluorescein (DCF) as previously described [32]. DCFH was added at a final concentration of 10 *μ*M and incubated for 30 min at 37°C. Cellular fluorescence was determined through flow cytometry (FACS-SCAN, Becton Dickinson, Franklin Lakes, NJ, USA). Subsequently, the cells were excited using an argon laser operating at 488 nM and measured at 510–540 nm.

### 2.9. Nrf2 Short Interfering (Si) RNA Transfection

Cardiac fibroblasts were transfected with either Nrf2 siRNA or control siRNA (obtained from Santa Cruz) by using the Lipofectamine reagent as described previously [31].

### 2.10. Statistical Analysis

Results were expressed as mean ± standard error of the mean (SEM) for at least three experiments unless otherwise stated. Statistical analysis was performed using Student's *t*-test or analysis of variance followed by Tukey's multiple comparisons using GraphPad Prism software (GraphPad Software, San Diego, CA, USA). *P* < 0.05 was considered statistically significant.

## 3. Results

### 3.1. Inhibitory Effect of Momordicine I on High-Glucose-Induced Cell Proliferation and Collagen Synthesis in Rat Cardiac Fibroblasts

Rat cardiac fibroblasts were treated with various concentrations of momordicine I, and cell viability was determined through MTT assay. The effects of momordicine I on cell viability in cultures are presented in Figure 1(b). Momordicine I had no toxic effect on rat cardiac fibroblasts at concentrations of 0.1–1 *μ*M, so concentrations of 0.1, 0.3, and 1 *μ*M momordicine I were used for further analysis. We subsequently tested the effect of momordicine I on cardiac fibroblast proliferation and collagen synthesis. Isolated cardiac fibroblast cells were cultured in normal- and high-glucose media. The effects of momordicine I on high-glucose-stimulated cardiac fibroblast proliferation and collagen synthesis were assessed by incorporating BrdU and ^3^H-proline. Compared with culturing in the normal-glucose medium, culturing in the high-glucose (25 mM) medium for 24 h slightly stimulated fibroblast proliferation and collagen synthesis (Figures 1(c) and 1(d)). Pretreatment of cardiac fibroblasts with momordicine I (0.3 and 1 *μ*M) for 12 h followed by exposure to a high-glucose medium resulted in a significant decrease in high-glucose-induced cell proliferation and collagen synthesis (Figures 1(c) and 1(d)). These data demonstrate that momordicine I inhibited high-glucose-induced cardiac fibroblast activation.

### 3.2. Inhibitory Effect of Momordicine I on High-Glucose-Induced TGF-*β*1 Secretion and Smad2/3 Phosphorylation in Rat Cardiac Fibroblasts

**Figure 2:**
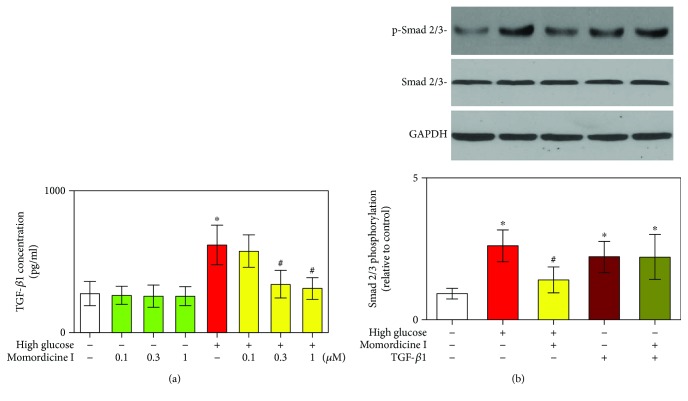
Effects of momordicine I on high-glucose-induced TGF-*β*1 secretion and Smad2/3 phosphorylation in rat cardiac fibroblasts. (a) Cardiac fibroblasts treated using a normal-glucose medium or high-glucose medium in the absence or the presence of momordicine I (0.1, 0.3, 1 *μ*M) for 24 h and the secretion of TGF-*β*1 in cardiac fibroblast supernatants measured through ELISA. Results are presented as mean ± SEM (*n* = 5). (b) Effect of momordicine I on high-glucose- or TGF-*β*1-induced Smad2/3 phosphorylation. The protein expression levels of Smad2/3 and p-Smad2/3 were detected using Western blotting following incubation with momordicine I (1 *μ*M) for 12 h; cardiac fibroblasts were exposed to high glucose for 24 h or TGF-*β*1 (10 ng/mL) for 2 h. Representative micrographs of Smad2/3 and p-Smad2/3 expression in Western blot analysis (upper) and quantitative results (lower). Results are presented as mean ± SEM (*n* = 3). ^∗^*P* < 0.05 versus the control group; ^#^*P* < 0.05 versus the high-glucose group.

To investigate whether momordicine I affects TGF-*β*1 in cardiac fibroblasts exposed to high-glucose medium, cardiac fibroblasts were treated with momordicine I under high-glucose conditions. TGF-*β*1 secretion was determined through ELISA. As depicted in Figure 2(a), the ELISA results revealed that the cardiac fibroblasts treated with high-glucose medium exhibited increased TGF-*β*1 secretion compared with the cardiac fibroblasts treated with a normal-glucose (5.6 mM) medium. However, high-glucose medium-induced TGF-*β*1 secretion was prevented by treating cardiac fibroblasts with momordicine I (0.3 and 1 *μ*M).

TGF-*β* receptor activation may increase Smad-2/3 phosphorylation and deploy many of their effects [20]. Therefore, phosphorylated Smad2/3 was also detected. Following incubation with momordicine I (1 *μ*M) for 12 h, cells were exposed to a high-glucose medium for 24 h or human-recombined TGF-*β*1 (10 ng/mL) for 2 h. After the aforementioned treatment, total cell proteins were extracted and subjected to Western blotting. As illustrated in Figure 2(b), levels of total Smad2/3 protein in cardiac fibroblasts remained unchanged after exposure to a high-glucose medium for 24 h or TGF-*β*1 for 2 h. However, the level of p-Smad2/3 was notably increased in high-glucose or TGF-*β*1-treated cardiac fibroblasts as compared to that in cells treated with a normal-glucose medium. Preincubation with momordicine I (1 *μ*M) partially but significantly suppressed high-glucose-induced phosphorylation of Smad2/3 protein. However, momordicine I (1 *μ*M) had no significant effect on TGF-*β*1-induced Smad2/3 phosphorylation. These observations indicated that momordicine I may inhibit the high-glucose-mediated fibrotic response by regulating the TGF-*β*1/Smad signaling pathway and suggested that momordicine I was able to suppress high-glucose-induced cardiac fibroblast activation through inhibition of TGF-*β*1 secretion, which was independent of its direct effect on the TGF-*β*1/Smad signaling pathway.

### 3.3. Antioxidant Effects of Momordicine I on High-Glucose-Induced ROS Production in Rat Cardiac Fibroblasts

**Figure 3:**
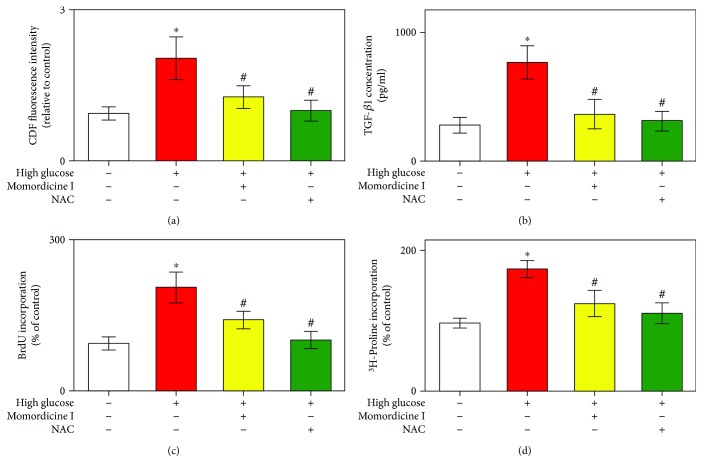
Momordicine I inhibits high-glucose-induced ROS in rat cardiac fibroblasts. (a) Effects of momordicine I on high-glucose-induced ROS generation. Cardiac fibroblasts were cultured in a normal-glucose medium or high-glucose medium for 30 min or preincubated with momordicine I (1 *μ*M, for 12 h) or NAC (5 mM, for 30 min) and then stimulated with the high-glucose medium for 30 min. Column bar graph of mean cell fluorescence for DCF. The fluorescence intensities in the control cells are expressed as 100%. (b) Effects of momordicine I and NAC on the high-glucose-induced secretion of TGF-*β*1 in cardiac fibroblasts. Effects of momordicine I and NAC on high-glucose-induced fibroblast proliferation (c) and collagen synthesis (d). Cardiac fibroblast cells were cultured in the control medium or high-glucose medium in the absence or presence of momordicine I (1 *μ*M) or NAC (5 mM) for 24 h. Results are presented as mean ± SEM (*n* = 4). ^∗^*P* < 0.05 versus the control group; ^#^*P* < 0.05 versus the high-glucose group.

Increased ROS plays a critical role in the development of DM-related cardiac fibrosis [4, 5, 11, 14, 15, 19]. We subsequently explored the role of momordicine I's antioxidant activity in the inhibition of the high-glucose-induced collagen synthesis. The ROS levels were detected from the fluorescence intensity of the ROS, which was analyzed using DCFH-DA assay and a flow cytometer [32]. Our data revealed that the fluorescence intensity enhanced by high glucose was significantly reduced by momordicine I (1 *μ*M) (Figure 3(a)). Notably, pretreatment with NAC (5 mM) for 2 h also substantially alleviated the ROS triggered by the high-glucose medium. As presented in Figure 3(b), cardiac fibroblasts treated with a high-glucose medium also exhibited increased TGF-*β*1 secretion compared with cardiac fibroblasts in the normal-glucose medium. However, high-glucose-induced TGF-*β*1 secretion was prevented by preincubation with momordicine I (1 *μ*M) or NAC (5 mM). Additionally, we analyzed the effect of momordicine I and NAC on cardiac fibroblast proliferation and collagen synthesis. Compared with the normal-glucose medium, the high-glucose medium stimulated fibroblast proliferation, which was significantly blocked by treatment with momordicine I or NAC (Figure 3(c)). In addition to the inhibition of the proliferative effect of the high-glucose medium on cardiac fibroblasts, momordicine I or NAC attenuated the collagen synthesis induced by the high-glucose-medium (Figure 3(d)). The effects of momordicine I on the high-glucose-induced ROS generation, TGF-*β*1 secretion, and fibroblast activation are similar to the effects of NAC, implicating its possible antioxidant role. These findings suggested that the antioxidant effect of momordicine I is related to the reduced oxidative stress induced by the high-glucose medium.

### 3.4. Activation of the Nrf2 Signaling Pathway by Momordicine I in Rat Cardiac Fibroblasts

**Figure 4:**
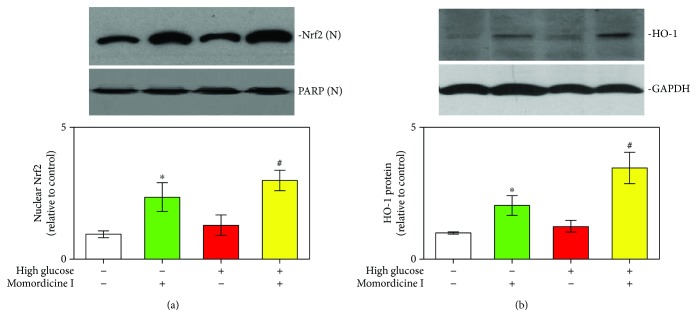
Momordicine I increases Nrf2 translocation and HO-1 protein expression in rat cardiac fibroblasts. (a) Effect of momordicine I on Nrf2 translocation. Cells were treated with or without momordicine I (1 *μ*M) for 12 h, followed by a normal-glucose medium or high-glucose medium for 12 h. N: nuclear extract. Results are presented as the mean ± SEM (*n* = 4). (b) HO-1 expression was determined through Western blotting. Cells were pretreated with momordicine I (1 *μ*M) or not for 12 h, followed by the control medium or high-glucose medium for 24 h. Results are presented as the mean ± SEM (*n* = 3). The relative protein expression of nuclear Nrf2 to PARP and HO-1 to GAPDH are presented in the bar graphs. ^∗^*P* < 0.05 versus the control group; ^#^*P* < 0.05 versus the high-glucose group.

The Nrf2/HO-1 signaling pathway is also associated with antifibrotic actions [33, 34]. To more thoroughly understand the possible signaling pathways and mechanisms in the action of momordicine I, we ascertained the expression of Nrf2 and Nrf2 downstream HO-1 in cardiac fibroblasts in high-glucose conditions. Our data revealed that a high-glucose condition slightly elevated the translocation of Nrf2 from the cytoplasm to the nucleus and the enhanced HO-1 expression; however, this modulatory effect was further augmented by momordicine I treatment (Figures 4(a) and 4(b)). These data implicated that momordicine I can activate the Nrf2/HO-1 signaling pathway in cardiac fibroblasts *in vitro*.

### 3.5. Nrf2 Inhibitor Brusatol or Nrf2 siRNA Abrogated the Inhibitory Effect of Momordicine I on High-Glucose-Induced TGF-*β*1 Secretion, Cell Proliferation, and Collagen Synthesis in Rat Cardiac Fibroblasts

**Figure 5:**
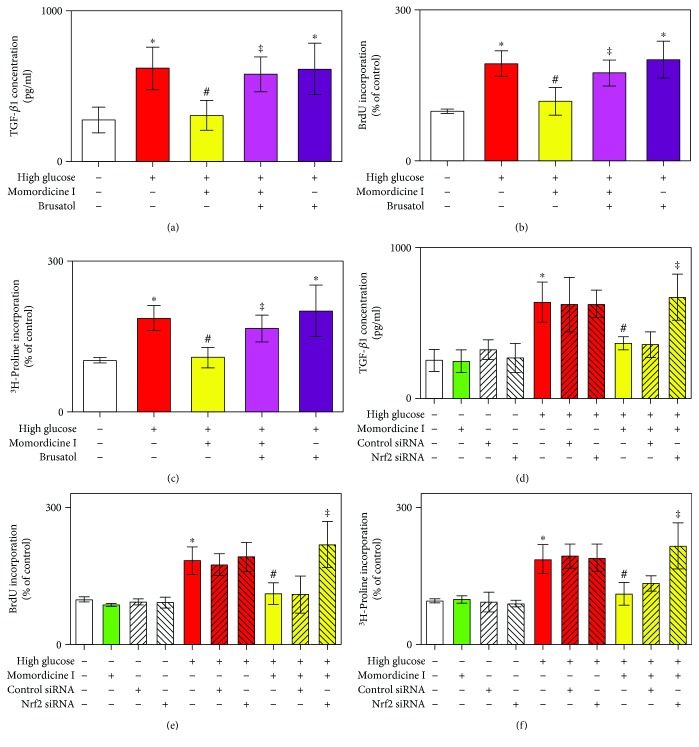
Momordicine I inhibits high-glucose-induced TGF-*β*1 secretion, cell proliferation, and collagen synthesis in rat cardiac fibroblasts in an Nrf2-dependent manner. (a) The Nrf2 inhibitor brusatol prevents the inhibitory effect of momordicine I on high-glucose-induced TGF-*β*1 secretion. Results are expressed as means ± SEM (*n* = 4). (b) Brusatol prevents the inhibitory effect of momordicine I on high-glucose-induced cell proliferation. Results are expressed as means ± SEM (*n* = 4). (c) Brusatol prevents the inhibitory effect of momordicine I on high-glucose-induced collagen synthesis. Cells were treated with brusatol (10 nM) for 30 min, followed by 1 *μ*M momordicine I for 12 h, and were subsequently cultured in high-glucose medium for 24 h. Results are expressed as means ± SEM (*n* = 4). (d) Nrf2 siRNA prevents the inhibitory effect of momordicine I on high-glucose-induced TGF-*β*1 secretion. Results are expressed as means ± SEM (*n* = 3). (e) Nrf2 siRNA prevents the inhibitory effect of momordicine I on high-glucose-induced cell proliferation. Results are expressed as means ± SEM (*n* = 3). (f) Nrf2 siRNA prevents the inhibitory effect of momordicine I on high-glucose-induced collagen synthesis. Results are expressed as means ± SEM (*n* = 3). ^∗^*P* < 0.05 compared with the control group; ^#^*P* < 0.05 versus the high-glucose group; ^‡^*P* < 0.05 versus momordicine I treatment in the high-glucose group.

To further verify the role of Nrf2 in the antifibrotic effect of momordicine I, brusatol, a specific Nrf2 inhibitor [35], was used to determine the role of Nrf2 in the effects of momordicine I on high-glucose medium-induced TGF-*β*1 secretion and cardiac fibroblast activation. Cells were treated with brusatol (10 nM, 30 min), followed by 1 *μ*M momordicine I for 12 h, and were subsequently cultured in a high-glucose medium for 24 h. The levels of TGF-*β*1 secretion significantly increased under high-glucose conditions compared with the normal-glucose control group (Figure 5(a)). However, these increases were inhibited by momordicine I. However, brusatol significantly abolished momordicine I's inhibitory effect on high-glucose-induced TGF-*β*1 secretion. As expected, momordicine I significantly reduced the high-glucose-induced fibroblast proliferation and collagen synthesis, which was also significantly reversed by brusatol (Figures 5(b) and 5(c)). The role of Nrf2 in the inhibition of high-glucose-induced TGF-*β*1 secretion, fibroblast proliferation, and collagen synthesis by momordicine I was also examined by silencing of Nrf2 (Figures 5(d)–5(f)). Cardiac fibroblasts transfected with Nrf2 siRNA (100 nM), followed by treatment with momordicine I (1 *μ*M) for 12 h, prevented the inhibitory effect of momordicine I on high-glucose-induced TGF-*β*1 secretion, fibroblast proliferation, and collagen synthesis. In contrast, the control siRNA (100 nM) failed to block the inhibitory effect of momordicine I. Collectively, these data indicated that momordicine I inhibited TGF-*β*1 pathway activation and proliferation and collagen synthesis of cardiac fibroblasts under a high-glucose condition in an Nrf-2 dependent manner.

## 4. Discussion

**Figure 6:**
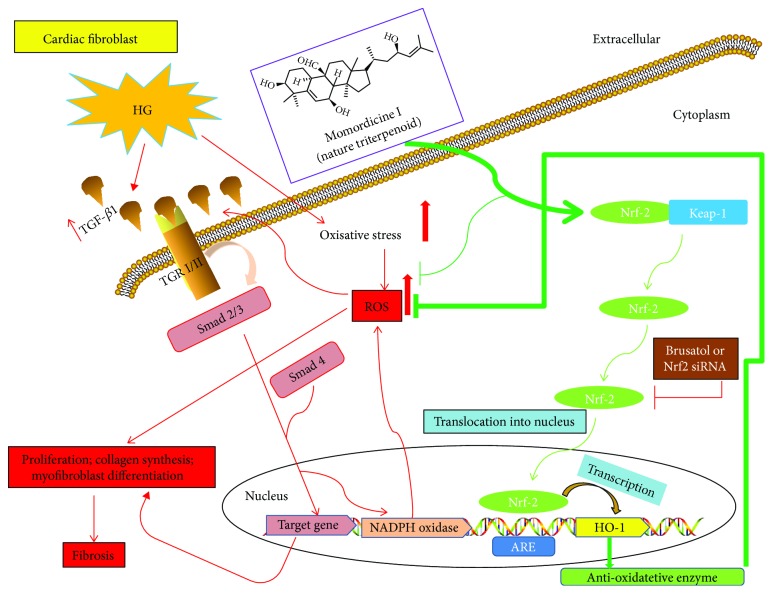
Schematic illustration of the proposed mechanism for high-glucose-induced rat cardiac fibroblast activation and momordicine I-inhibited fibrogenesis through the Nrf2-mediated inhibition of oxidative stress and TGF-*β*1 signaling.

Through a series of *in vitro* experiments, we determined that a high-glucose condition led to an increase in cell proliferation, collagen synthesis, and the expression of TGF-*β*1 and Smad2/3 phosphorylation in cardiac fibroblasts. Momordicine I pretreatment significantly reduced the high-glucose-induced fibroblast activation by inhibiting the TGF-*β*1-Smad2/3 signaling pathway. We further demonstrated that momordicine I can ameliorate high-glucose-induced ROS by activating the Nrf2/HO-1 pathway. Furthermore, brusatol (an Nrf2 inhibitor) or Nrf2 siRNA abrogated the inhibitory effect of momordicine I on high-glucose-induced TGF-*β*1 secretion. These results suggested that momordicine I inhibits fibrogenesis through the Nrf2-mediated modulation of TGF-*β*1-Smad2/3 signal transduction (Figures 6).

Consumption of food containing antioxidants has been revealed to protect against diseases such as cancer, cardiovascular diseases, and DM [25, 36]. *Momordica charantia*, commonly known as bitter melon, is a climbing perennial characterized by an elongated, warty fruit-like gourd and has been reported to have medicinal qualities in treating DM [25, 30, 37–39]. A principal therapeutic constituent of bitter melon known as momordicine has long been used in traditional Asian medicine [30]. Momordicine I belongs to cucurbitane-type triterpenoids, the major bioactive components of bitter melon. These have anticancer, antioxidant, antidiabetic, hypoglycemic, and anti-inflammatory properties [27, 30, 39–41]. With literature review and our study, we suppose that momordicine I may be used as complementary treatment targeting cardiac fibrosis in patients with DM.

The pathophysiological mechanisms of DCM are multifactorial; substantial evidence from both clinical data and animal models has indicated that increased cardiac inflammation, oxidative stress, and enhanced cardiac fibrosis contribute to the development of DCM [42]. Therefore, anti-inflammatory, antioxidant, and antifibrotic therapeutic approaches may be beneficial for treating DCM. Fibrogenesis is the most critical factor in the progression of DCM. However, specific pharmaceuticals directly targeting fibrosis are still lacking. In this study, despite clearly acting as an antifibrotic result, momordicine I has been shown to demonstrate antioxidant effects. Furthermore, extensive evidence has indicated that oxidative stress mediates the initiation and development of fibrosis in relation to chronic inflammation [18]. Although our main focus was the modification of ROS levels as a mediator of downstream high-glucose-induced TGF-*β*1 signaling [20], we discovered that momordicine I alleviated the production of hyperglycemia-induced ROS and might block the activation of high-glucose-induced cardiac fibroblast by inhibiting ROS and its downstream TGF-*β*1 signal. Additionally, studies have indicated that TGF-*β*1 increases ROS generation by inducing NADPH oxidase and suppressing antioxidant enzymes; in turn, ROS activates TGF-*β*1 signaling and mediates several of its fibrogenic effects, in a vicious cycle [2, 15, 18, 20]. These results further strengthen the evidence for the antioxidant properties of momordicine I through its inhibition of ROS. To confirm the role of oxidative stress in high-glucose-induced cardiac fibroblast activation, we treated cardiac fibroblasts with the antioxidant NAC. Similarly, the ROS scavenger NAC also decreased high-glucose-induced cardiac fibroblast activation. However, whether any antioxidant could have the beneficial effects of momordicine I in decreasing high-glucose-induced cardiac fibroblast activation remains to be examined in future studies.

The TGF-*β* family of growth factors is the most extensively studied mediator of fibroblast activation, of which TGF-*β*1 likely plays a crucial role in pathological fibrosis [9]. The profibrotic actions of TGF-*β*1 on cardiac fibroblasts are mediated, at least partially, by Smad3 [20, 43, 44]. Bujak et al. demonstrated that the loss of Smad3 prevents interstitial fibrosis in the noninfarcted remodeling of the myocardium [44]. TGF-*β*1/Smad signaling appears to be responsible for cardiac fibrosis [20]. The canonical pathway of TGF-*β*1 signaling involves the phosphorylation of Smad2/3, which subsequently binds to Smad4 and translocates to the nucleus. Here, transcriptional reprogramming is conducted to promote myofibroblast formation and extracellular matrix production, eventually leading to cardiac fibrosis [9, 20, 45]. Our paper suggests that momordicine I at nontoxic concentrations (0.1–1 *μ*M) effectively attenuates the profibrogenic TGF-*β*1-Smad2/3 signaling pathway and is thus an effective therapy for diabetes-associated cardiac fibrosis. Several natural compounds, including matrine, resveratrol, and tanshinone, also suppressed cardiac fibrosis by inhibiting the TGF-*β*/Smad pathway [46–48].

Under normal conditions, Nrf2 is retained within the cytoplasm, forming a complex with its protein inhibitor Keap1 [36]. On activation, Keap1 cysteine residues undergo oxidoreduction resulting in the release of Nrf2, which subsequently translocates into the nucleus where it binds to ARE and controls the transcription of genes encoding antioxidant enzymes [49]. Nrf2 governs innate immune, antioxidant, and cytoprotective responses, and its deregulation is pivotal in the chronic inflammatory status [33]. Nrf2 has long been recognized to resist oxygen-free radicals and reduce ROS in the alleviation of cardiac fibrosis [34]. A study reported that the forced expression of Nrf2 in normal fibroblasts resulted in the abrogated stimulation of collagen synthesis, myofibroblast differentiation, and ROS generation through the disruption of canonical TGF-*β*1 signaling [33]. Our study revealed that momordicine I not only ameliorates high-glucose-induced ROS but also effectively promotes Nrf2 translocation to the nucleus, which in turn upregulates HO-1. In this study, by using a selective Nrf2 inhibitor or Nrf2 knockdown, we confirmed that the antifibrotic effects of momordicine I on cardiac fibroblasts under high-glucose conditions were Nrf2 dependent. He et al. investigated the role of Nrf2 in the development of DCM using Nrf2-knockout mice. There was an increased level of ROS in the cardiomyocytes of Nrf2-knockout mice [50]. Notably, Nrf2 attenuated dystrophic muscle fibrosis by inhibiting the TGF-*β*1/Smad pathway [51]. Our study demonstrated that brusatol or Nrf2 siRNA significantly reversed the inhibitory effect of momordicine I on high-glucose-induced fibroblast activation and TGF-*β*1 expression. Thus, momordicine I might reduce high-glucose-induced TGF-*β*1 expression, cell proliferation, and collagen synthesis through the direct antifibrotic effects of Nrf2 and indirect downregulation of ROS levels. However, the exact molecular mechanism of Nrf2-mediated gene regulation in cardiac fibroblasts under high-glucose conditions by momordicine I remains unknown and warrants further investigation. Moreover, the induction of HO-1 is widely recognized as an effective cellular strategy to counteract a variety of cellular damage and inflammation. These effects may be mediated by multiple functions of HO-1. Although the exact mechanisms involved in the antifibrotic effects of HO-1 have not been fully elucidated, momordicine I, as confirmed in this study, can induce the expression of HO-1 in cardiac fibroblasts; the observed antifibrotic effects of momordicine I might be mediated, at least in part, by one or more of HO-1 by-products. However, whether inhibition of HO-1 activity could block momordicine I-induced antifibrotic action to suggest the role of HO-1 on the antifibrotic effect of momordicine I in cardiac fibroblasts remains to be elucidated.

A limitation of our study is that the antifibrotic efficacy of momordicine I was determined using a single TGF-*β*1-dependent *in vitro* fibrosis model. Furthermore, the momordicine I-reduced high-glucose-induced collagen production from its inhibitory effect on proliferation cannot be excluded. The exact mechanism and potential role of momordicine I in fibroblast collagen synthesis remain to be investigated. The present *in vitro* model may not be directly translatable to clinical investigations of DCM. However, our preliminary results may encourage further research on determining the molecular mechanisms underlying high-glucose-induced fibroblast activation and cardiac fibrosis. While these results are promising, a lot of work needs to be done in elucidating the signal pathways involved in the action of momordicine I on these cells. Moreover, *in vivo* experiments in animal models are essential to establish the validity of these *in vitro* results in the future.

## 5. Conclusions

On the basis of this study, we propose that through Nrf2 activation, momordicine I can partially block intracellular TGF-*β*1 signal transduction and inhibit the proliferation and collagen synthesis of cardiac fibroblasts. Through this activity, momordicine I reduces collagen synthesis and TGF-*β*1 expression induced by high-glucose treatment, suggesting its potential for clinical application in preventing and treating diabetic myocardial fibrosis. Our study demonstrated that momordicine I attenuates high-glucose-induced fibroblast activation and its antifibrotic effects, at least partially, appear to reduce the expression of the profibrogenic cytokine TGF-*β*1 and inhibit TGF-*β*1-Smad2/3 signaling. This suggests that momordicine I has therapeutic potential in treating diabetes-induced cardiac fibrosis.

## Data Availability

The data of materials and methods and conclusions to support the findings of this study are included within the article. If any other data may be needed, please contact the corresponding author upon request.
