# Corrigendum to “Inhibitory Effects of Momordicine I on High-Glucose-Induced Cell Proliferation and Collagen Synthesis in Rat Cardiac Fibroblasts”

**DOI:** 10.1155/2019/2514095

**Published:** 2019-04-04

**Authors:** Po-Yuan Chen, Neng-Lang Shih, Wen-Rui Hao, Chun-Chao Chen, Ju-Chi Liu, Li-Chin Sung

**Affiliations:** ^1^Department of Biological Science and Technology, College of Biopharmaceutical and Food Sciences, China Medical University, Taichung 40402, Taiwan; ^2^Department of Life Sciences, College of Science, National University of Kaohsiung, Kaohsiung 811, Taiwan; ^3^Division of Cardiology, Department of Internal Medicine, Shuang Ho Hospital, Taipei Medical University, New Taipei City 23561, Taiwan; ^4^Division of Cardiology, Department of Internal Medicine, School of Medicine, College of Medicine, Taipei Medical University, Taipei 11031, Taiwan

In the article titled “Inhibitory Effects of Momordicine I on High-Glucose-Induced Cell Proliferation and Collagen Synthesis in Rat Cardiac Fibroblasts” [1], there was an error in the fourth affiliation where “Division of Cardiology” was missing. The corrected affiliation is shown above.
